# Treatment and therapeutic strategies for pituitary apoplexy in pregnancy: a case series

**DOI:** 10.1186/s13256-021-02892-5

**Published:** 2021-05-27

**Authors:** Yuya Kato, Yoshikazu Ogawa, Teiji Tominaga

**Affiliations:** 1 Department of Neurosurgery, Tohoku University Graduate School of Medicine, Kohnan Hospital, 4-20-1 Nagamachiminami, Taihaku-ku, Sendai, Miyagi 982-8523 Japan; 2 Department of Neurosurgery, Kohnan Hospital, Sendai, Miyagi Japan

**Keywords:** Lactotroph cell adenoma, Pituitary apoplexy, Pregnancy, Transsphenoidal surgery

## Abstract

**Background:**

Pregnancy is a known risk factor for pituitary apoplexy, which is life threatening for both mother and child. However, very few clinical interventions have been proposed for managing pituitary apoplexy in pregnancy.

**Case presentation:**

We describe the management of three cases of pituitary apoplexy during pregnancy and review available literature. Presenting symptoms in our case series were headache and/or visual disturbances, and the etiology in all cases was hemorrhage. Conservative therapy was followed until 34 weeks of gestation, after which babies were delivered by cesarean section with prophylactic bolus hydrocortisone supplementation. Tumor removal was only electively performed after delivery using the transsphenoidal approach. All three patients and their babies had a good clinical course, and postoperative pathological evaluation revealed that all tumors were functional and that they secreted prolactin.

**Conclusions:**

Although the mechanism of pituitary apoplexy occurrence remains unknown, the most important treatment strategy for pituitary apoplexy in pregnancy remains adequate hydrocortisone supplementation and frequent hormonal investigation. Radiological follow-up should be performed only if clinical symptoms deteriorate, and optimal timing for surgical resection should be discussed by a multidisciplinary team that includes obstetricians and neonatologists.

## Introduction

Pituitary apoplexy (PA) is a rare but potentially life-threatening condition caused by rapid enlargement of a pituitary adenoma secondary to intratumoral hemorrhage and/or infarction [1–3]. Presenting symptoms are sudden onset of headache, visual impairment, ophthalmoplegia, and reduced consciousness due to adrenal insufficiency [4]. Pregnancy increases the risk of PA, and PA in a pregnant woman is associated with significant risk of death for both mother and child. PA in pregnancy has been successfully treated in a few cases, but no major clinical evidence for treatment strategy has been described [5]. There are a few reports about therapeutic options to restart or increase dopamine agonist medication for prolactin-producing adenomas during the pregnancy itself [5, 6]; however, this option should be carefully considered because dopamine agonists are also risk factors for PA occurrence. Here, we report successful treatment of PA in three pregnant patients and discuss treatment and therapeutic strategies for PA in pregnancy.

## Case presentation

### Case 1

**Fig. 1:**
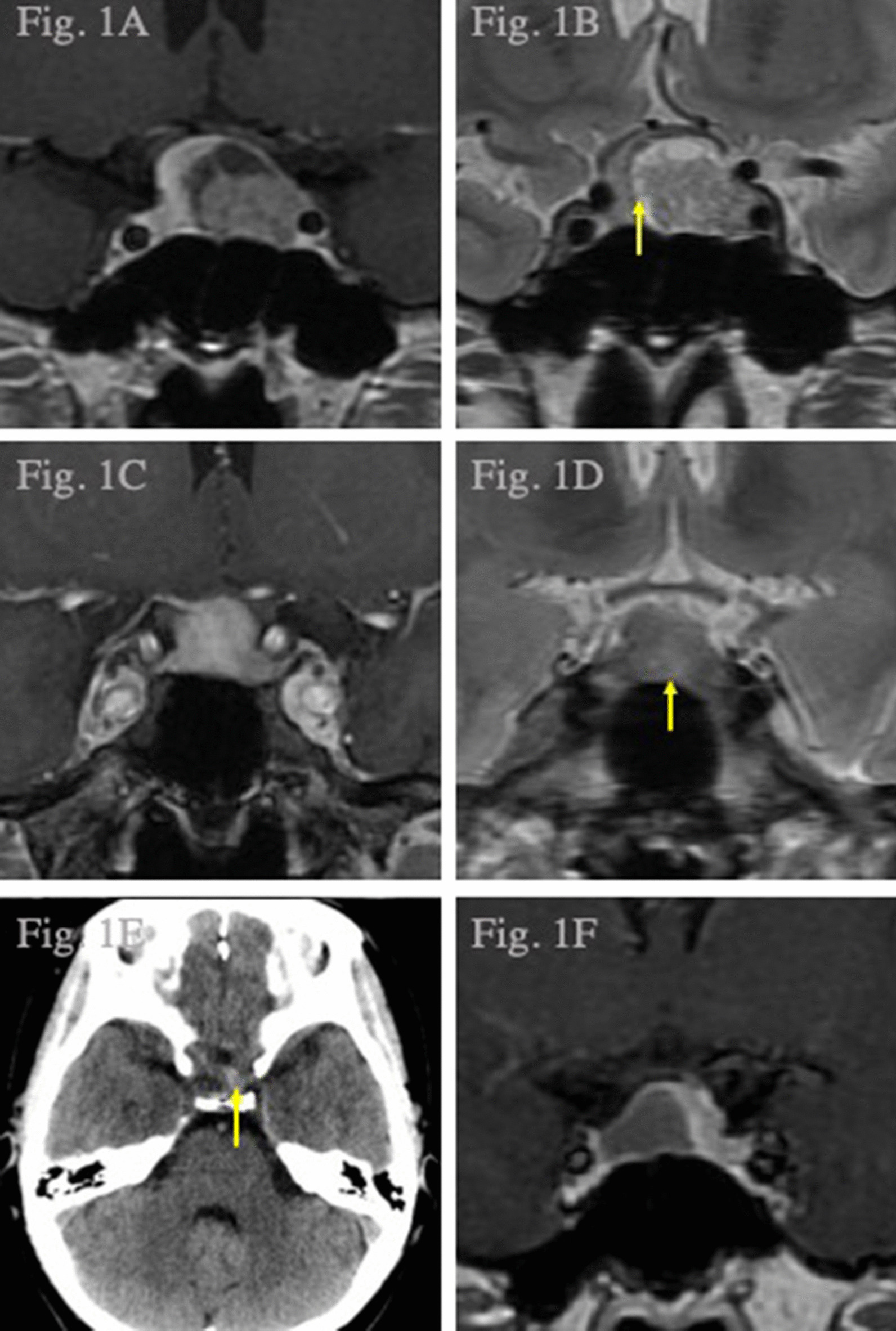
Coronal T1-weighted MR images of the head with contrast medium (case 1, **a**). Coronal T2-weighted head MR image; yellow arrow shows the hematoma in the tumor (case 1, **b**). Coronal T1-weighted head MR images with contrast medium (case 2, **c**). Coronal T2-weighted head MR image with the yellow arrow showing the hematoma in the tumor (case 2, **d**). Computed tomography scan of the head with the yellow arrow showing hemorrhage in the sella turcica (case 3, **e**). Coronal T1-weighted head MR images with contrast medium (case 3, **f**) showing a large sellar tumor that was compressing the optic chiasm anteriorly

A 33-year-old Japanese woman presented with secondary amenorrhea and infertility despite no family history of cancer or endocrinological diseases. Hyperprolactinemia (368 ng/mL, normal range 4.9–29.3 ng/mL) was detected 3 years after her first delivery, and terguride (1 mg/day) was prescribed; however, it was stopped when a second pregnancy was confirmed. Additionally, left temporal hemianopia was detected when she was admitted to the gynecology department for imminent abortion at 35 weeks of gestation (WG). Magnetic resonance (MR) imaging of the head with contrast medium revealed a large sellar tumor (15 × 16 × 15 mm) with irregular enhancement that was compressing the optic chiasm upwards (Fig. 1a, b). Except hyperprolactinemia, all other hormone levels were within the normal range, and she was referred to our department for further management. The neonatology treatment team recommended waiting for 1 week before performing a cesarean section with a prophylactic bolus supplementation of hydrocortisone, and this approach resulted in the birth of a healthy child. Transsphenoidal surgery was performed 2 weeks later, and the tumor could be completely removed. Hydrocortisone (200 mg/day) was started as hormonal replacement therapy; the dose was gradually reduced and changed to dexamethasone (0.5 mg/day). She was discharged without deficits. The patient showed no clinical signs of hypopituitarism during 3 months after surgery, and dexamethasone was stopped after confirming normal basal hormone levels. Postoperative pathological examination of the tumor identified the tissue as a lactotroph adenoma with a number of Ki-67 positive cells that was not very high at 68 per three high-power fields.

### Case 2

A 22-year-old pregnant Japanese woman presented with headache and foggy visual field defects at 32 WG, and she had no family history of cancer or endocrinological diseases. Right temporal hemianopia and a large sellar tumor (13 × 14 × 10 mm) upon imaging were detected, and the latter, which was compressing the optic chiasm anteriorly, showed irregular enhancement with contrast medium (Fig. 1c, d). An endocrinological evaluation revealed only hyperprolactinemia (55 ng/mL) and no other abnormalities, and she was referred to our department. The neonatology treatment team recommended a waiting period of 2 weeks before performing a cesarean section with prophylactic bolus supplementation of hydrocortisone, which resulted in the delivery of a healthy child. Transsphenoidal surgery was performed 2 weeks later, and the tumor was totally resected. Her postoperative course was uneventful, hydrocortisone was started as hormonal replacement therapy after surgery, and the dose was gradually reduced and changed to dexamethasone. She was discharged without deficits. The patient showed no clinical signs of hypopituitarism during 3 months after the procedure, and dexamethasone was ceased after confirming normal basal hormone levels. Postoperative pathological examination of the tumor revealed a plurihormonal adenoma that was secreting prolactin and follicle-stimulating hormone-β but with a very low number of Ki-67 positive cells (17 per three high-power fields).

### Case 3

A 29-year-old pregnant Japanese woman was admitted to the emergency department with sudden onset of severe headache at 28 WG. She had no family history of cancer or endocrinological diseases, and computed tomography revealed slight hemorrhage in the sella turcica (Fig. 1e). MR imaging of the head revealed a large sellar tumor (8 × 13 × 11 mm) that was anteriorly compressing the optic chiasm, and the tumor showed irregular enhancement upon imaging with contrast medium (Fig. 1f). An endocrinological panel was normal except for hyperprolactinemia (192 ng/mL), and she was referred to our department. The neonatology treatment team recommended waiting for 9 weeks, but she complained of repeated headaches during the waiting period, which required frequent acetaminophen use. Nevertheless, 9 weeks later, a cesarean section was performed with prophylactic bolus supplementation of hydrocortisone, and she delivered a healthy child. Transsphenoidal surgery was performed 1 year later for the enlarging tumor, and the tumor was completely removed. Her postoperative course was uneventful, and hydrocortisone was started as hormonal replacement therapy after surgery. The dose was gradually reduced and changed to dexamethasone. She was discharged without deficits. The patient showed no clinical signs of hypopituitarism during 3 months after surgery, and dexamethasone was stopped upon confirmation of normal basal hormone levels. Pathological analysis of the tumor revealed a lactotroph adenoma with a very low number of Ki-67 positive cells (3 per three high-power fields).

## Discussion

Several risk factors for PA have been identified, including tumor factors such as histological type, nonfunctioning tumor or prolactinoma, and size (macroadenoma) [2], along with patient factors such as pregnancy, dopamine agonist administration, systematic hypertension, dynamic pituitary function tests, and anticoagulant agents [2, 5, 7]. Pregnancy may induce PA through two possible mechanisms. First, the pituitary gland increases in volume in pregnant women compared with nonpregnant women [8], and second, as estrogen receptors are expressed in the lactotroph cells, estrogen levels become very high during pregnancy, and these lactotroph cells undergo massive hyperplasia. Therefore, even though lactotroph cells become larger, both in the pituitary gland and the pituitary adenoma, blood supply remains limited [6]. Additionally, dopamine agonists used for the treatment of prolactinoma could induce PA because they promote tumor volume reduction and degeneration or necrosis, which result in intratumoral hemorrhage [9].

Only a few reports have described the management of PA in pregnancy, and of these, in a retrospective summary of 17 such cases, 10 cases were treated surgically and 7 cases were treated conservatively with dopamine agonists and hydrocortisone. Moreover, two cases in the surgical group were administered bromocriptine before surgery for the immaturity of the fetus [6]. Previous strategies described for the management of PA, both before and during pregnancy, did not recommend routine radiological follow-up during pregnancy, and MR imaging was typically limited to the patients with clinical suspicions of acute changes. In contrast, strict evaluation of the thyrotroph and corticotroph axes is critical during each trimester, along with monitoring T4 and urinary free-cortisol levels. Furthermore, overall hormonal work-up is crucial for evaluating PA, and adequate hydrocortisone therapy should be the first intervention [5, 6].

In our study, all patients could sustain the pregnancy despite the occurrence of PA, and the babies were delivered by cesarean section at 34 WG to minimize hemodynamic fluctuations, because alveolar surfactant formation is sufficiently complete by this time. However, this is contingent on the pregnant woman's medical condition as well. Although our patients underwent elective transsphenoidal tumor removal after delivery, conversely, there are a few reports about therapeutic options to restart or increase dopamine agonist medication during the pregnancy itself [5, 6]; however, again, this option should be carefully considered because dopamine agonists are also risk factors for PA occurrence. Additionally, such risk evaluation should depend on both medical and surgical interventions and close relationships. If PA occurs in pregnancy, endocrinological evaluation and adequate hydrocortisone supplementation should be planned, followed by delivery and subsequent transsphenoidal surgery. Although most patients have had a favorable outcome, a few patients have suffered severe outcomes such as cardiac arrest [8].

## Conclusions

Although the underlying mechanism of PA development remains unknown, the most important treatment option for PA in pregnancy remains adequate hydrocortisone supplementation and frequent hormonal work-up. Radiological follow-up should be performed only if clinical symptoms deteriorate. Last, the optimal time for surgical resection should be discussed in a multidisciplinary team comprising obstetricians and neonatologists.

## Data Availability

Because this manuscript is a case report, there are no relevant datasets that were used to support the conclusions stated in this article.

## Abbreviations

PA: Pituitary apoplexy
MR imaging: Magnetic resonance imaging
